# Cellular metabolic activity marker via selective turn-ON detection of transporter protein using nitrobenzoxadiazole-based fluorescent reporter

**DOI:** 10.1038/s41598-020-60954-y

**Published:** 2020-03-05

**Authors:** Tanoy Dutta, Kaushik Pal, Apurba L. Koner

**Affiliations:** 1 Department of Chemistry, Indian Institute of Science Education and Research Bhopal, Bhopal Bypass Road, Bhauri, Bhopal, 462066 (MP) India; 2 Present Address: Department of Physics and Astronomy, Iowa State University, lowa, IA 50011 USA

**Keywords:** Biophysical chemistry, Small molecules, Diagnostic markers, Fluorescence spectroscopy

## Abstract

A nitrobenzoxadiazole-based fluoroprobe (NBD-Bu) is designed to probe cellular metabolic activity in cancer and normal cells. NBD-Bu shows a significant fluorescence enhancement upon selective binding to the transport protein serum albumin in PBS buffer at ambient conditions. Encouraged by this finding, the site- specificity of NBD-Bu has been explored through a competitive displacement assay in the presence of site-specific markers such as warfarin and ibuprofen. Notably, even at micromolar concentrations, the probe possesses the ability to displace the site marker drug ibuprofen, efficiently. Subsequently, high-resolution fluorescence imaging results consolidated the potential of NBD-Bu for detection of abnormal cellular metabolic activity.

## Introduction

Serum albumin (SA) is the most abundant water-soluble protein found in blood plasma occupying almost 60% of total blood plasma proteins^1,2^. Human serum albumin (HSA) has 585 amino acid residues in a single polypeptide chain, while bovine serum albumin (BSA), which is 76% homologous to HSA, has 583 amino acid residues^1^. It functions as a versatile transport protein, transporting small drug molecules, bile-salt, hormones, vitamins, metals and plays a significant role by contributing 80% to the maintenance of oncotic pressure between blood vessels and tissues^3,4^. Moreover, albumins are primarily responsible for maintaining the pH of the blood^5,6^. SA is also used as an additive in the cell culture media as it enhances growth and cell viability^7^. A high level of SA is triggered by severe dehydration and high protein diet while deficiency of the same causes dysfunction of the circulatory system, which inclines the detection of SA to be extremely important^6^.

Among the vastly employed and reported techniques in this purpose, cyclic voltammetry^8,9^, circular dichroism spectroscopy^10^, nuclear magnetic resonance spectroscopy^11,12^, high-performance liquid chromatography, absorption spectroscopy, and mass spectrometry are quite useful. However, these methods are not as popular as fluorescence spectroscopy is because of their sophistication, and poor sensitivity. Fluorescence spectroscopy is one of the widely used and efficient tools to study the interaction between drug molecules and SA because of its rapidness, good selectivity, as well as high sensitivity and especially real-time visualization by naked-eye^13,14^. Several fluorescent dyes are already known for SA binding, but the binding site and stoichiometry is not explored in most of the cases^14–17^. Quite a few numbers of fluorescent probes have been developed based on their sensitivity towards local polarity and viscosity, which causes a significant change in emissive states upon binding to the multiple hydrophobic pockets present in SA^18^. Nevertheless, limitations arise due to the absorbance of the probes in the near-UV region resulting in the interference by the auto-fluorescence of protein molecules, change in the secondary structure of protein upon ligand binding and lower selectivity of the sensor while other relevant analytes are present^19^. Nitrobenzoxadiazole (NBD) dyes are well explored in the literature for their interesting fluorogenic intramolecular charge-transfer (ICT) properties and utilized for sensing, and protein binding studies^20^. NBD-labeled lipids are well-explored fluorescent probes for understanding membrane structure and dynamics, and they have been widely used in both living cells and model systems^21,22^.

## Results and Discussion

The Nitrobenzoxadiazole (NBD) moiety is involved with the easy synthetic procedure and it is well-known for its sensitivity towards environment polarity, thereby being a potential sensor for micellar as well as proteinaceous microenvironments. Based on previous reports, a short butyl chain was incorporated in the NBD moiety to impart better fluorescence property and appropriate lipophilicity for improved binding affinity with the hydrophobic pocket of the protein^23^. Following the synthesis of NBD-Bu, it was characterized by NMR spectroscopy (see SI, Figs. S1–2) and mass spectrometry (Fig. S3). The optical purity was also confirmed by comparing the absorption and excitation spectra (Fig. S4). NBD-Bu contains an electron-rich nitrogen atom, tethered to a butyl chain and an electron-deficient NO_2_ group. These two moieties are linked *via π*-conjugated linker, which renders it to be a classical ICT dye. In general, ICT dyes are well responsive to the solvent polarity thereby exhibiting polarity-dependent photo physical behavior^24^.

**Scheme 1:**
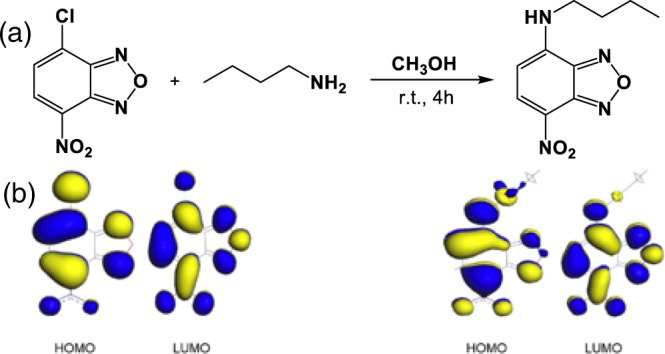
(**a**) Synthetic scheme of the fluoro probe N-butyl-7-nitrobenzo[c][1,2,5]oxadiazol-4-amine (NBD-Bu), (**b**) frontier molecular orbital picture of NBD moiety before and after substitution shows ICT properties.

**Figure 1:**
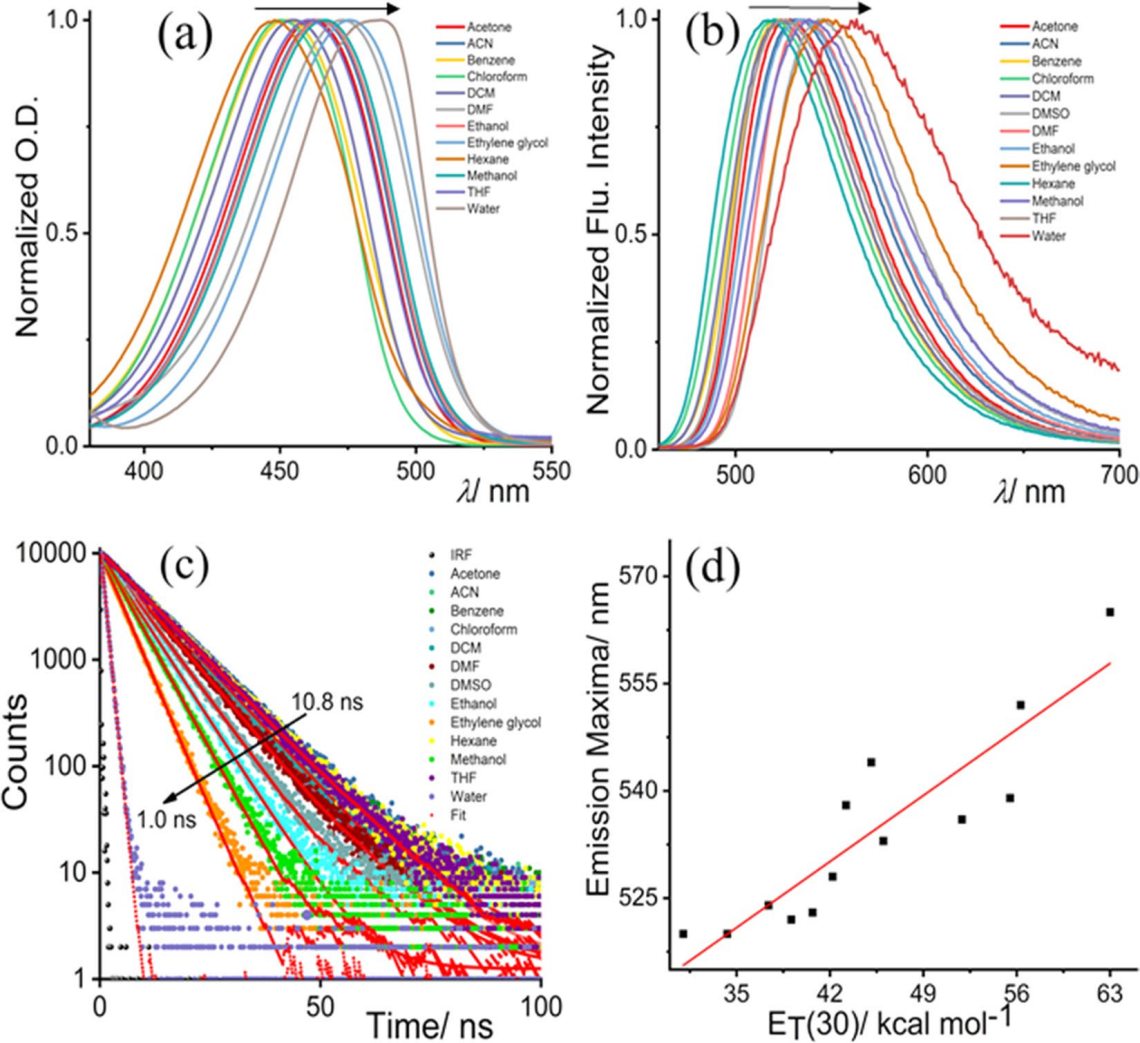
Solvent-dependent photophysical properties of NBD-Bu: (**a**) normalized UV-Vis. spectra with the varying solvent polarity, (**b**) normalized fluorescence spectra, (**c**) time-resolved fluorescence decay profile and (**d**) emission maxima vary linearly (*R*^2^ = 0.82) with solvent polarity parameter. Arrows indicate the direction of low to high solvent polarity.

To validate the ICT properties of NBD-Bu (Scheme 1), we have performed a thorough spectroscopic investigation using different solvents with varying polarity. In both ground and excited states, the spectra shifted towards longer wavelength along with spectral broadening upon increasing solvent polarity (Fig. 1a,b, Table S1, SI). Subsequently, the fluorescence lifetime of NBD-Bu (Fig. 1c) was found to be longer in the non-polar solvents compared to polar ones, which are mainly due to the presence of hydrogen bonding interaction between dye and solvent molecules originating from higher non-radiative decay rates in polar solvents. The emission maxima and fluorescence lifetime varied linearly (Figs. 1d, S5) with polarity consolidating the fact that NBD-Bu can be used for local environment sensing. Such a feature makes it an excellent candidate to sense the local micro-polarity inside the hydrophobic pockets of protein molecules.

**Figure 2:**
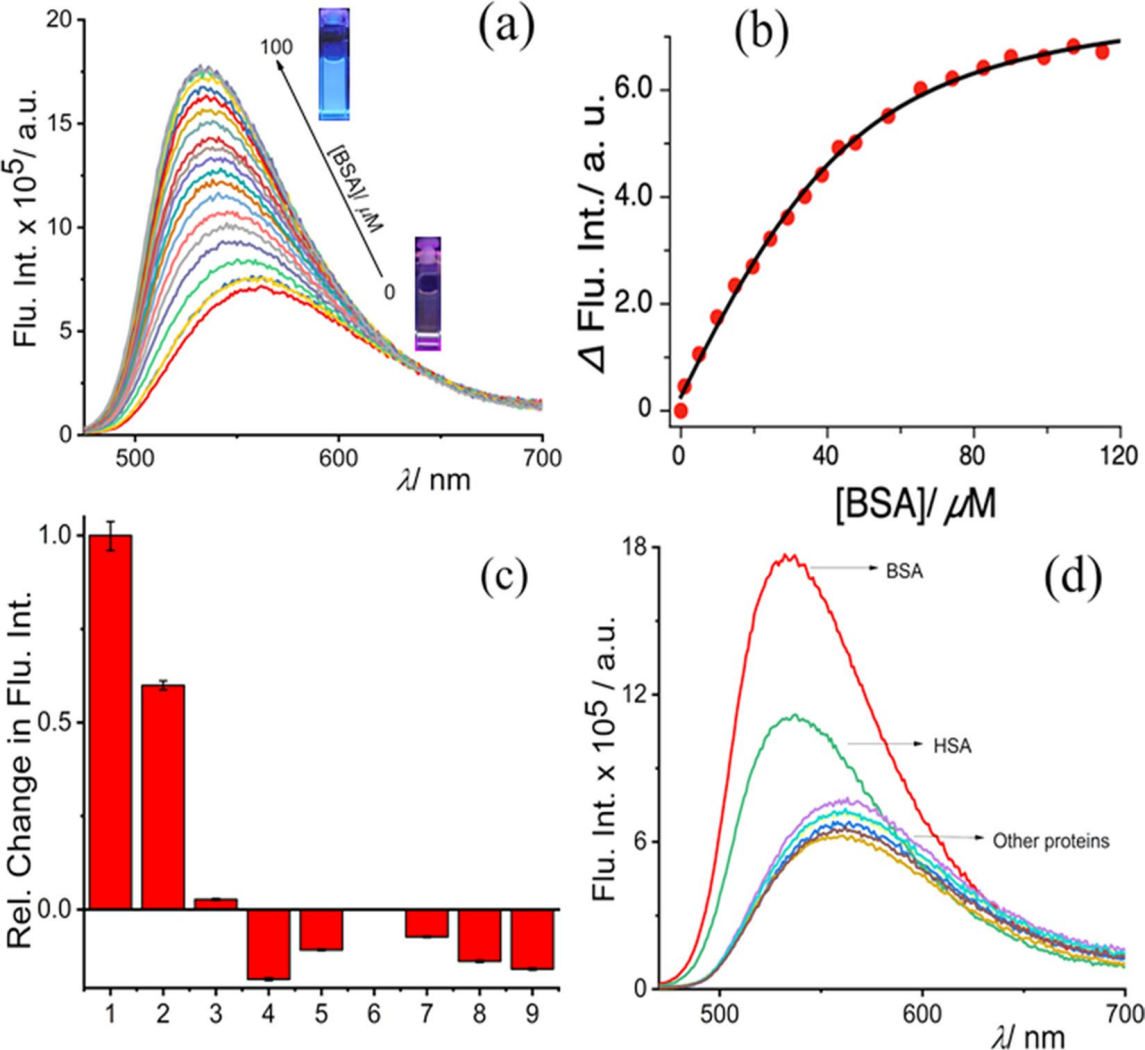
Fluorescence titration of NBD-Bu (10 *µ*M) with increasing BSA (up to 100 *µ*M) concentration (**b**) plot of fluorescence intensity of NBD-Bu with BSA concentration fitted with 1:1 binding equation, (**c**) relative change in the fluorescence intensities of NBD-Bu (10 *µ*M) in presence of other biologically relevant proteins (100 *µ*M): 1. BSA, 2. HSA, 3. Trypsin, 4. Lysozyme, 5. *α*-amylase, 6. *α*-Chymotrypsin, 7. RNAse, 8. DNAse and 9. *α*-lactalbumin, (**d**) the global analysis of fluorescent spectra of NBD-Bu with all proteins in PBS (pH 7.4).

Considering the excellent solvatochromic property of NBD-Bu, we studied its absorption and fluorescence response for assessing hydrophobic pockets of proteins in PBS (pH 7.4). NBD-Bu possesses good water solubility as well as excellent pH stability in the range of pH 2–9 (SI, Figs. S6–9). The probe itself is weakly emissive in the aqueous medium; however, its fluorescence intensity increased gradually upon increasing concentration of BSA along with a significant 30 nm blue-shift without affecting the absorption maxima (SI, Fig. 2a, S10 and Table S1). The emission maxima of NBD-Bu bound BSA was similar to the neat acetone which supports the previous report from Kudo *et al*.^25^. Upon addition of BSA, the fluorescence intensity increased thrice, instantaneously, triggering the propensity of detecting BSA in an expeditious manner.

This interesting result inspired us to investigate further the binding phenomena of NBD-Bu with BSA. As small molecules with similar van der Waals volume are known to bind with SA in 1:1 mode^20^, hence, in this case, the binding curve was fitted (Fig. 2b) using the with 1:1 binding equation. The result showed a remarkably high binding constant, K_a_ = 8.3 × 10^5^ M^−1^. The limit of detection (LOD) was calculated to be 0.22 *µ*M (Fig. S11). Further, isothermal titration calorimetry (ITC) experiments were performed (Figs. S12–14) to determine the thermodynamic parameters for BSA/NBD-Bu binding and the displacement assay of ibuprofen by NBD-Bu (see Table S2). The results are well in accordance with the steady-state binding studies. The binding of NBD-Bu was further verified with fluorescence lifetime and time-resolved anisotropy analysis (Figs. S15–17). Furthermore, the selectivity, another important aspect that defines the applicability of a sensing probe, was assessed. In this regard, our probe was found to be non-responsive towards other physiologically relevant proteins with structural and functional diversity (Fig. 2c,d).

**Figure 3:**
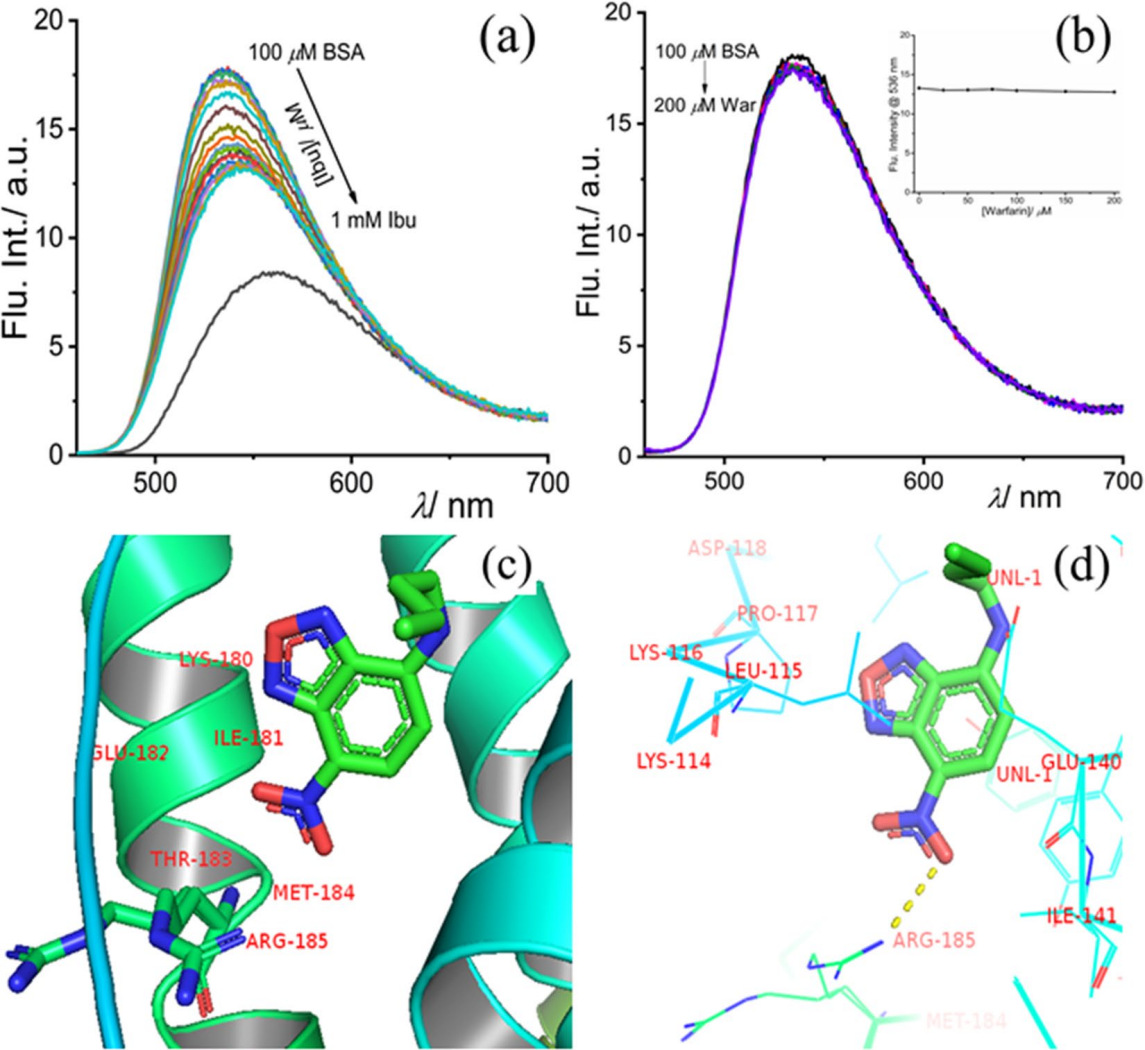
Competitive binding assay of NBD-Bu (10 *µ*M), pre-incubated with 100 *µ*M BSA, with increasing concentration of (**a**) ibuprofen and (**b**) warfarin in PBS (pH 7.4); (inset: the intensity at 535 nm with increasing site-marker concentration) (**c,d**) molecular docking picture shows the binding interaction of NBD-Bu with BSA. The molecular docking result was analyzed by PyMOL (v2.1.1) and UCSF Chimera (v1.13, https://www.cgl.ucsf.edu/chimera/) for visualization and understanding.

The slight interference in the presence of HSA can be attributed to its structural similarity with BSA. Hence, the selective behavior of the probe can be well acclaimed. Following up on the binding studies of the probe with BSA, the site-specificity within the hydrophobic pocket of the protein molecule was investigated. Ibuprofen and warfarin are well-explored drug molecules known to bind specifically to site-II and site-I, respectively. The competitive binding experiments (Fig. 3a,b) suggest that ibuprofen could replace the bound dye only to a certain extent indicating that NBD-Bu binds to the same site as that of ibuprofen and has a stronger binding affinity, too. This proves that NDB-Bu specifically binds to site-II of BSA without affecting its secondary structure (Fig. S18). Even 1 mM ibuprofen could only reduce the intensity up to ~25% along with a 10 nm red-shift. Interestingly, warfarin showed no effect upon similar treatment and the fluorescence intensity remained unchanged.

To gain further insight into these phenomena, we have performed molecular docking studies and analyzed by PyMOL, which were consistent with competitive binding experiments with site-selective markers (Fig. 3c). NBD-Bu structure, optimized using Gaussian 09, was further used for all docking studies. The stabilization energy (ΔG_*Binding*_ = ΔG_*vdW*_ + ΔG_*H-bonding*_ + ΔG_*Electronic*_ + ΔG_*Conformational*_ + ΔG_*Torsional*_ + Δ[(ΔG_*Water*−_ΔG_*Site*−*II*_)_*NBD*–*Bu*_] of BSA bound NBD-Bu was found to be −7.32 kcal/mol. Such high binding and stabilization energy is originated due to multiple weak intermolecular interactions such as hydrogen bonding, hydrophobic interaction present between NBD-Bu and the side-chain present in the vicinity of site-II (as shown in Fig. 3d).

After a detailed understanding of the specificity and binding phenomena of NBD-Bu with BSA, we investigated the validity and applicability of our findings using fluorescence microscopy. For this purpose, initially, the MTT assay was performed in four different cell lines (both cancerous and non-cancerous, see Fig. S19, SI) and IC_50_ value was found to be hovering around 50 *µ*M. Whereas, more than 70% of the cells were viable when incubated with 10 *µ*M dye concentration confirming the suitable working concentration for further cell imaging studies. Interestingly, NBD-Bu was found to localize mostly in the endoplasmic reticulum (see Figs. S20–22, SI) that has been comparatively studied with commercially available organelle markers.

**Figure 4:**
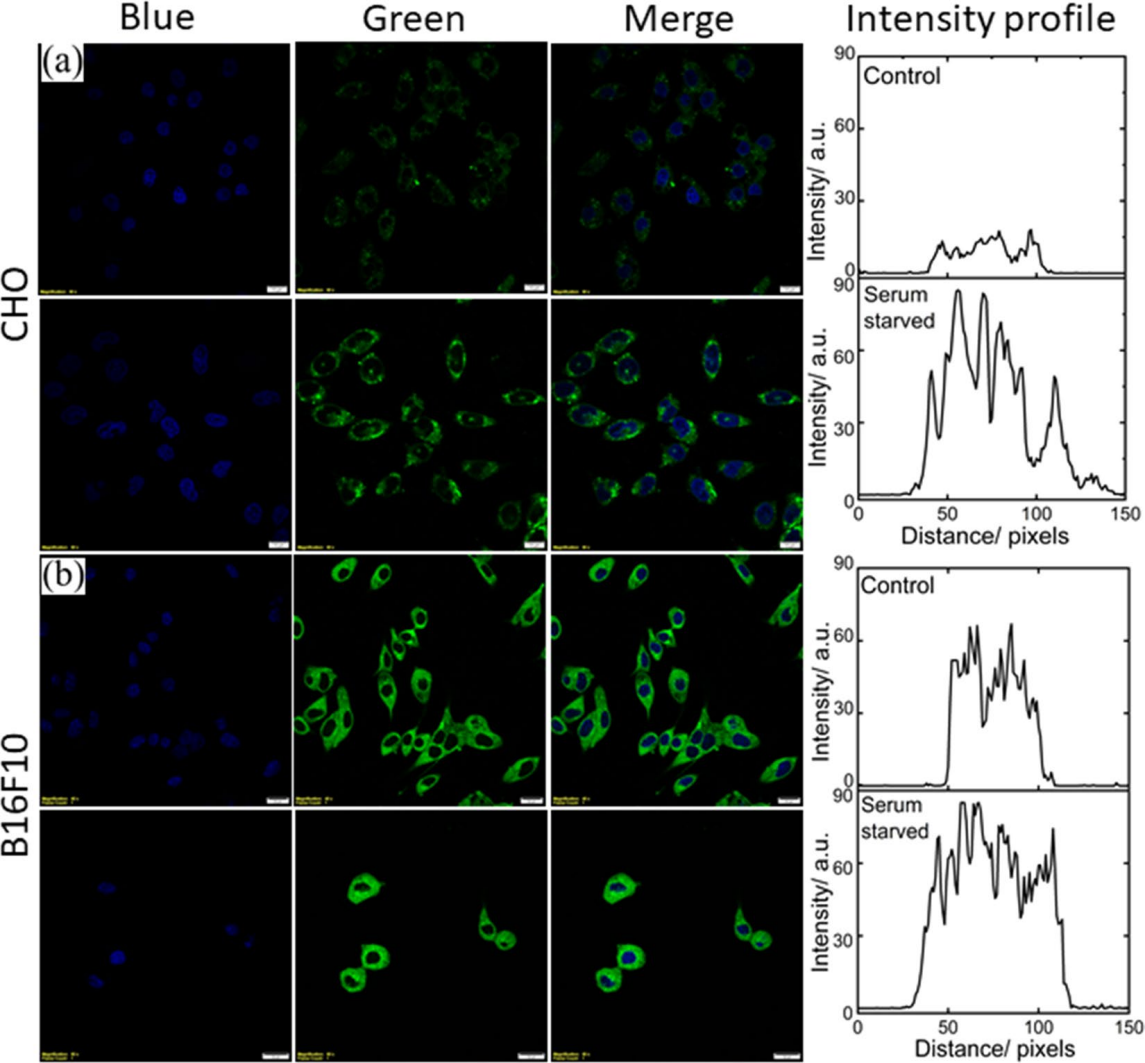
Live-cell confocal microscopy imaging of (**a**) CHO (scale bar 20 *µ*m) and (**b**) B16F10 (scale bar 5 *µ*m) cell lines. The left blue panel is for nuclear staining using Hoechst 33342; middle panel for the green channel for NBD-Bu and right is merged of the blue and green channel. The corresponding average intensity obtained from the line profiles shows much higher fluorescence enhancement with serum-starved cells compared to untreated ones.

Recent report^26^ suggests an important role of abnormal metabolism during cancer resulting in the disease itself, rather than uncontrolled cell proliferation. Therefore, different normal (CHO, and BHK-21; see top panel of Fig. 4 for CHO and Fig. S23 for BHK-21) and cancer cell lines (B16F10, and HeLa; see bottom panel of Fig. 4 for B16F10 and Fig. S24 for HeLa) have been taken to distinguish the metabolic activity using NBD-Bu as a reporter. The CHO and B16F10 cells were grown for 24 h to reach 70% confluency followed by 6 h of serum-starvation. Finally, they were incubated for 20 min with 10 *µ*M NBD-Bu for imaging. The confocal microscopy images of CHO cells and B16F10 cells (Fig. 4a,b) show that the fluorescence intensity is considerably higher in the serum-starved cells (followed by 100 *µ*M BSA treatment) compared to the well-fed cells. This is evident from the intensity profiles obtained from the corresponding green channel images. The intensity in serum-starved CHO cells is ~4 times to that of untreated ones. On another hand, the intensity is only ~1.5 times in starved B16F10 cells compared to well-fed cells. This can be attributed to the higher uptake capacity of cancer cells due to higher metabolic activity compared to normal cells, which resulted in greater intensity even in the well-fed cells. This argues towards the applicability of NBD-Bu as a metabolic marker to visualize the difference in metabolism in normal and cancer cells.

**Figure 5:**
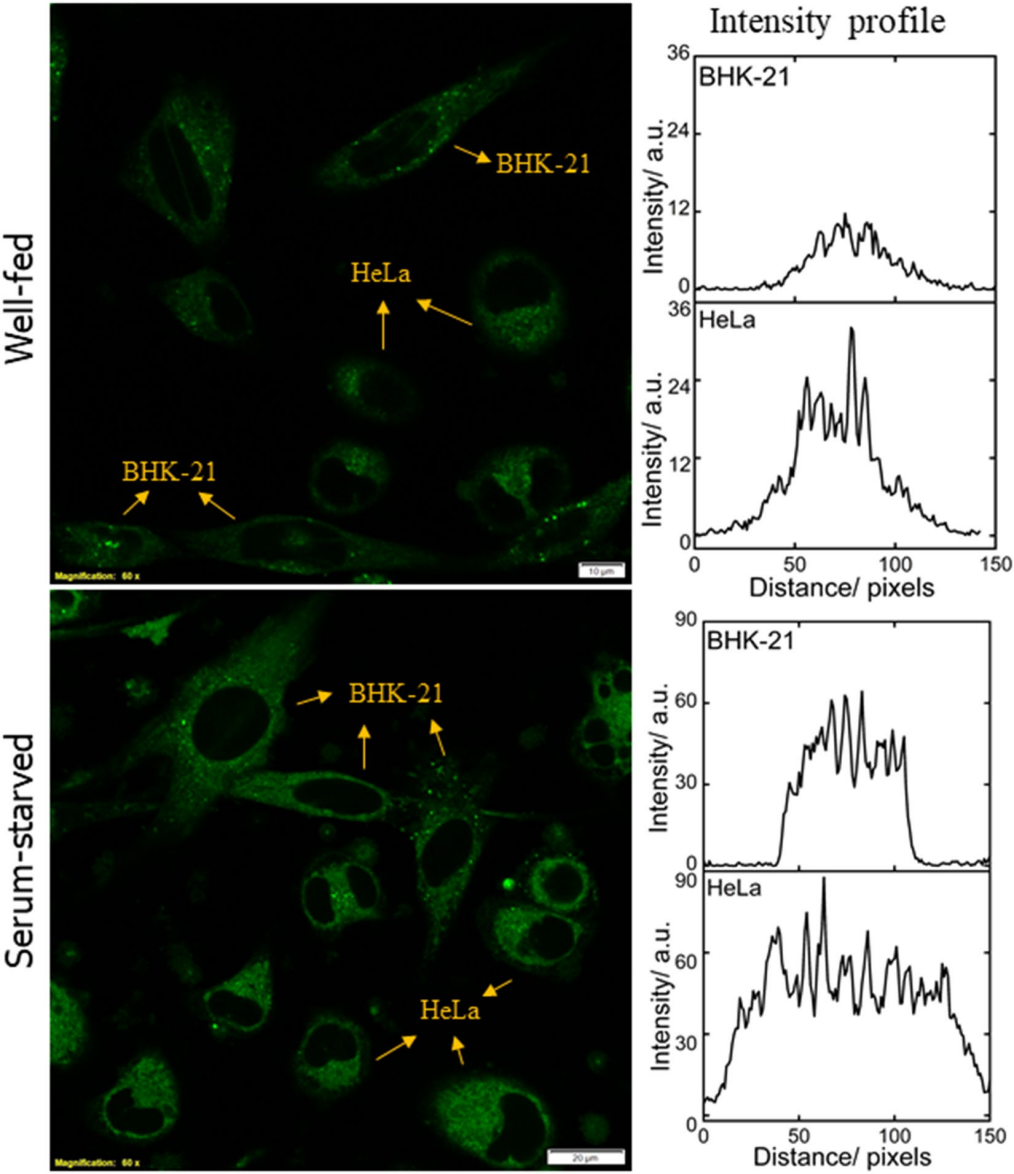
Live-cell confocal microscopy imaging of BHK-21 and HeLa cell lines in co-culture condition, (**a**) well-fed and (**b**) serum-starved cells and their corresponding intensity profile (averaged from 4 ROIs of each cell lines per image) shows the greater cellular uptake in cancer cells. Scale bar 20 *µ*M.

The results obtained from the monoculture of normal and cancer cell lines were further verified with co-culture conditions (Fig. 5). BHK-21 and HeLa cells were seeded in 1:1 ratio and grown for 48 h followed by 6 h of serum-starvation. Serum-starved cells were additionally incubated with 100 *µ*M BSA for 30 min before further incubation for 20 min with 10 *µ*M NBD-Bu. Living up to our anticipation, the intensity recorded in well-fed HeLa cells was ~3.5 times compared to BHK-21 cells, whereas only ~1.5 times higher intensity was measured in serum-starved HeLa cells (Fig. 5, right panel). These observations are well in synchronization with the monoculture results (*vide supra*).

In summary, we have designed an NBD-based intramolecular charge transfer reporter for instantaneous and efficient detection of serum albumin, one of the important transport proteins in the presence of other biologically relevant proteins. The detailed spectroscopic studies revealed that the probe is binding to site-II of BSA and was further supported by the competitive binding and molecular docking studies. Finally, employing the binding phenomena, the difference in metabolic activity in non-cancerous and cancer cells have been well elucidated by the live-cell microscopic investigation in monoculture and co-culture conditions which showed that serum-starvation led to the enhancement of intensity in cancer cells compared to normal cells. We believe that this probe can be employed for the detection of abnormal serum albumin levels in the patient’s body fluids and as metabolic activity marker for diagnostics applications.

## Experimental Section

### Chemicals and materials

4-Chloro-7-nitrobenzofurazan, warfarin, trypsin (from bovine pancreas), *α*-Chymotrypsin (from bovine pancreas) and *α*-lactalbumin (from bovine milk) were purchased from Sigma-Aldrich (India). n-Butyl amine was purchased from Spectrochem (India). HSA, RNase A (from bovine pancreas, free from DNase), Catalase (from the bovine liver), *α*-Amylase and DNase (from bovine pancreas) were purchased from HIMEDIA (USA). BSA was purchased from BIOMATIK, USA. All the solvents were of spectroscopic grade and were used without any further purification. Milli-Q grade water was used for the spectroscopic measurements with a resistivity of 18.2 M*Ω*·cm at 298 K.

### Synthesis and characterization

^1^H and ^13^C NMR spectra were recorded on Bruker 400 MHz spectrometers with operating frequencies of 100 MHz for ^13^C using CDCl_3_ as solvent and tetramethylsilane (TMS) as an internal standard. Chemical shifts (*δ*) are reported in ppm relative to the residual solvent signal (*δ* = 7.26 for ^1^H NMR and *δ* = 77.3 for ^13^C NMR). HRMS data were recorded on MicrOTOF-Q-II mass spectrometer using Acetonitrile as a solvent.

To a solution of 4-Chloro-7-nitrobenzofurazan (100 mg, 0.501 mmol) in 10 mL methanol, butylamine (99.03 *µ*L, 1.002 mmol) was added dropwise over the course of 5 minutes. The reaction was performed in an inert atmosphere for 5 hours at room temperature. Then the solvent was removed under reduced pressure. The residue was purified by silica gel column chromatography using 30–40% mixture of EtOAc and Hexane as eluent and the yield was calculated to be 60%. ^1^H NMR (400 MHz, CDCl_3_) *δ*: 8.5 (1H, d, J = 8.6 Hz), 6.18 (1H, d, J = 8.7 Hz), 3.5 (2H, q, J = 6.7 Hz), 1.8 (2H, m, J = 7.3 Hz), 1.51 (2H, dt, J = 14.8, 7.5 Hz), 1.02 (3H, t, J = 7.3 Hz). ^13^C{^1^H} NMR (400 MHz, CDCl_3_) *δ*: 144.26, 143.88, 136.50, 123.98, 98.50, 43.71, 30.54, 20.14, 13.68. HRMS (SI) m/z [M+ H]^+^ calcd. for C_10_H_12_N_4_O_3_ 237.0909 Da; found 237.0982 Da.

### Spectroscopic measurements

Steady-state absorption spectra were recorded on a double-beam Cary 5000 UV-spectrophotometer. All steady-state fluorescence measurements were performed using a spectrofluorimeter (model: Fluorolog-3) from HORIBA Jobin Yvon. All spectroscopic data were recorded by using a quartz cuvette having 1 cm path length and 4.8 cm height with *λ*_ex_ = 450 nm. Both excitation and emission slit width were kept at 1 nm while recording the emission spectra of NBD-Bu. Time-resolved fluorescence measurements were performed using a Hamamatsu MCP photomultiplier (R-3809U-50). The time-correlated single photon counting (TCSPC) setup consists of an Ortec 9327 pico-timing amplifier and using pulse Diode laser (*λ*_ex_ = 470 nm) with fwhm ~143 ps with a setup target 10,000 counts. The emission polarizer was positioned at the magic angle (54.7°) polarization w.r.t. the excitation polarizer. The single exponential fitting function was employed by iterative deconvolution method using supplied software DAS v6.2 as described earlier^27,28^. The quality of the fitted data was judged from the reduced chi-squared value (*χ*^2^), calculated using the IBH software provided with the instrument. Circular dichroism spectra were measured using JASCO J-815 CD spectrometer with standard sensitivity (100 mdeg), 0.1 nm data pitch with scanning speed 200 nm min^−1^.

### Optimization and molecular docking studies

The structure of NBD-Bu was optimized with Gaussian 09 with method RB3LYP/6–31G (Calculation type: FOPT). The molecular docking studies have been done with SwissDock^29^, based on the EADock DSS engine, a web server for docking of small molecules on the target proteins. The PDB file of BSA (4F5S) and the optimized structure were used for the docking. The job was submitted to the server and obtained results were analyzed by PyMOL (v2.1.1) and UCSF Chimera^30^ (v1.13) for visualization and understanding.

### Isothermal titration calorimetry (ITC) experiments

The thermodynamic parameters for NBD-Bu/BSA, Ibu/BSA and NBD-Bu/Ibu-BSA binding interactions were studied by isothermal titration calorimetric (ITC) experiments using a Nano ITC from TA instrument. The ITC measurements were performed at 298 K. For an ITC experiment, a degassed NBD-Bu solution (50 *μ*L,150 *μ*M) was injected into the cell containing 300 *μ*L of the 35 *μ*M BSA solution using a syringe at a rotating speed of 250 rpm. The time interval between two injections was kept at 200 s. Control experiments were implemented using a 10 mM PBS buffer (pH 7.4) and NBD-Bu to get the heat change of dilution. The ITC results were analyzed using the NanoAnalyze v3.10.0 software. An independent site-binding model was used for fitting to estimate the thermodynamic parameters. According to the power convention of the NanoAnalyze v3.10.0, the exothermic processes are shown by upward heat burst curves. The negative free energy changes (ΔG < 0) indicate thermodynamically favorable process.

### Cell culture and imaging

#### Materials and microscopy

Dulbecco’s Modified Eagle Medium (DMEM) and antibiotic cocktail were purchased from HiMedia (USA) and Fetal Bovine Serum (FBS) was purchased from Sigma Aldrich (USA). The cell imaging dishes were obtained from ibidi (Germany). All the confocal microscopy imaging was performed with an Olympus FV3000 Confocal Laser Scanning Microscope (LSM). The image processing was done with the help of cellSens software (Olympus).

#### Culture method

B16F10, HeLa, CHO and BHK-21 cells were obtained from National Center for Cell Science, Pune, India and cultured in DMEM (phenol red free) containing 10% (v/v) FBS and 1% (v/v) antibiotic cocktail in 5% CO_2_ at 37 °C in incubator. For imaging purposes, cells were grown to 75–80% confluency in the 35 mm glass-bottom imaging dishes (170 ± 5 *µ*m). For co-culture experiment, BHK-21 and HeLa cells were seeded in 1:1 ratio and grown for 48 h followed by 6 h of serum-starvation. Serum-starved cells were additionally incubated with 100 *µ*M BSA for 30 min before further incubation for 20 min with 10 *µ*M NBD-Bu.

#### Serum starvation experiment

The growth medium was removed and washed twice with sterilized PBS buffer (pH 7.4, containing 5 mM MgCl_2_). Then the cells were incubated with only DMEM (serum-free) for 6 h. Thereafter, the cells were washed gently with PBS and further incubated with 100 *µ*M BSA for 15 min at 37 °C. After washing them again with PBS, all the cells, starved and non-starved ones, were incubated together with 8 *µ*M Hoechst 33342 for nuclear staining and 10 *µ*M NBD-Bu for 20 min at 37 °C. Finally, they were washed and imaged under confocal LSM.

#### Confocal microscopy

For fluorescence imaging, 405 nm (for Hoechst 33342 from ThermoFisher Scientific, USA) and 488 nm (for NBD-Bu) excitation lasers were used. For 405 nm and 488 nm excitation, the emission windows were kept at 430–470 nm and 500–600 nm, respectively. The confocal aperture was kept at 0.81 Airy Disk (AU) while the dwell time is 10 *µ*s/pixel. The laser power, gain and offset were kept the same for all. The colocalization microscopic experiments have been performed with 5 or 10 *µ*M NBD-Bu and 0.3 *µ*M commercial tracker dye incubating for 20 minutes at 37 °C in the CO_2_ incubator. The emission window was kept as 500–540 nm and 570–650 nm for the excitation of 488 nm (2% of laser power) and 561 nm (0.5% of laser power), respectively. The images were acquired in sequential scan mode which ensures that the two fluorophores are not excited simultaneously.

#### Image analysis

The post-processing was performed using ImageJ (Fiji) software^31^ (v 1.52n). The line profiles of the selected ROIs were plotted as grayscale intensity vs. distance. These values were averaged to finally obtain an intensity profile.

## Supporting information

Supporting information.
