# Supplementary material for: Cellular metabolic activity marker via selective turn-ON detection of transporter protein using nitrobenzoxadiazole-based fluorescent reporter
Source: Sci Rep. 2020 Mar 5;10:4166. doi: 10.1038/s41598-020-60954-y (PMC7058046; doi:10.1038/s41598-020-60954-y)
Supplement: Supplementary file 1 — Supporting information. [file 41598_2020_60954_MOESM1_ESM.pdf]

Supporting Information

Cellular metabolic activity marker via selective turn-ON detection of transporter protein using nitrobenzoxadiazole-based fluorescent reporter

Tanoy Dutta,[‡] Kaushik Pal,[†‡] and Apurba L. Koner*[‡]

[‡] Department of Chemistry, Indian Institute of Science Education and Research Bhopal, Bhopal Bypass Road, Bhauri, Bhopal 462066 (MP), INDIA, [†] present address: Department of Physics and Astronomy, Iowa State University, IA-50011, USA. * Corresponding Author: E-mail: akoner@iiserb.ac.in

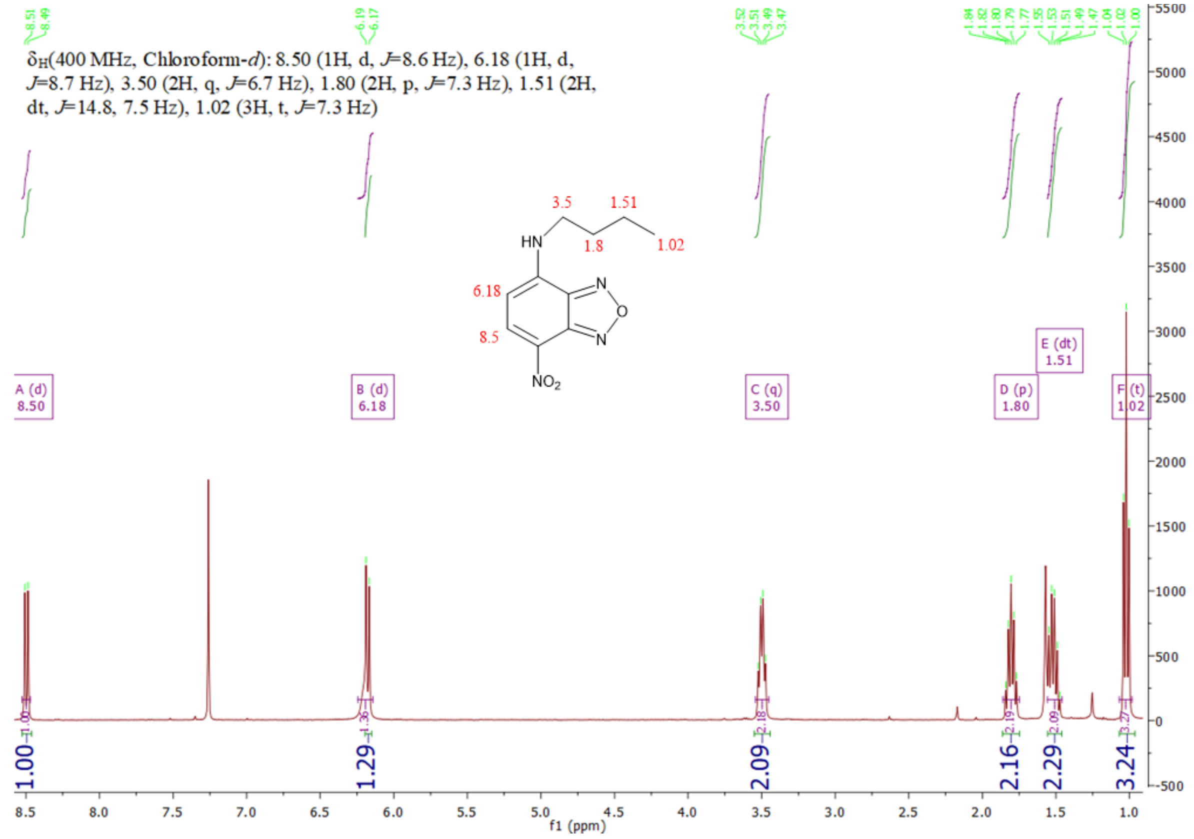

Fig. S1: ^{1}H NMR spectra (400 MHz) of NBD-Bu in $CDCl_3$

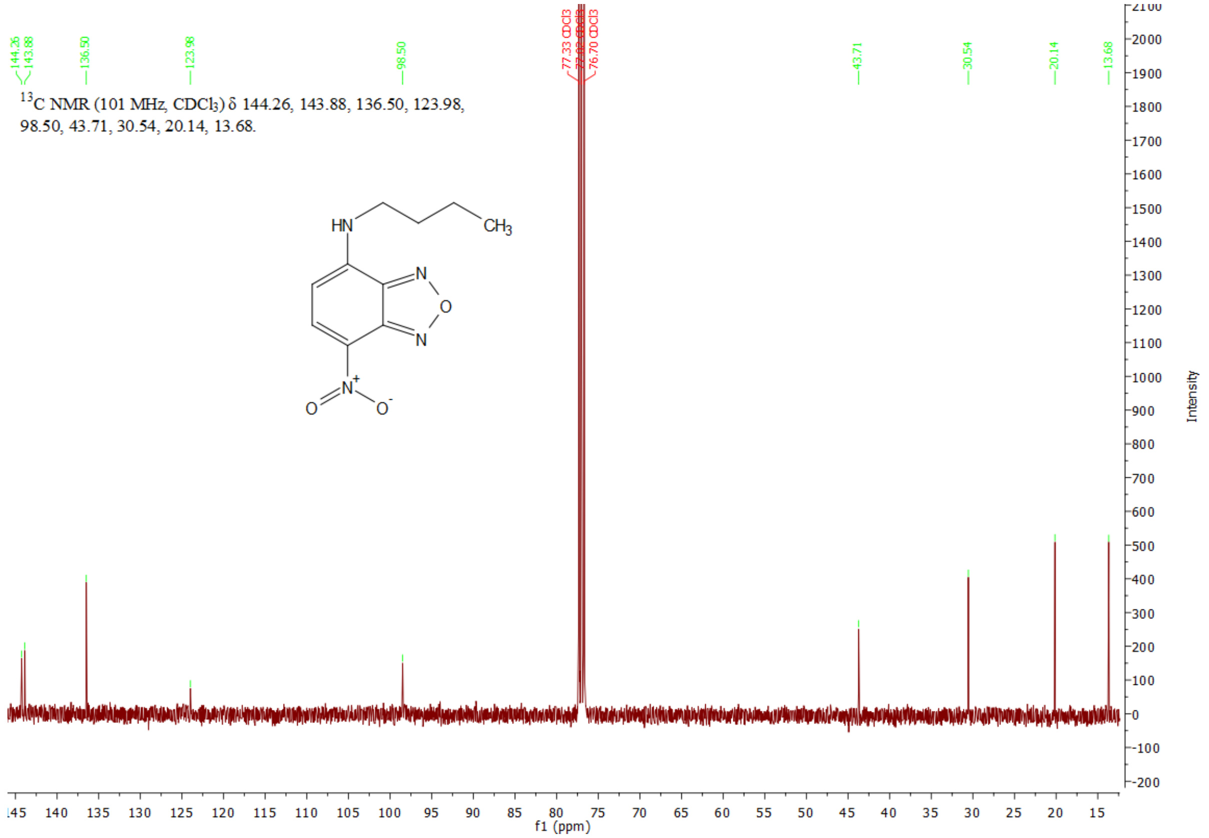

Fig. S2: $^{13}C\{^{1}H\}$ NMR spectra (126 MHz) of NBD-Bu in $CDCl_3$

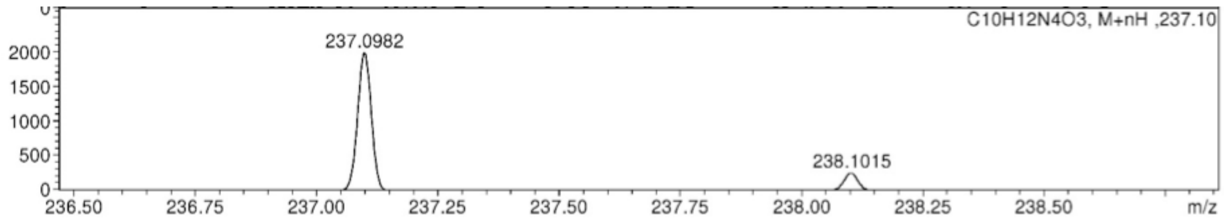

Fig. S3: High-resolution mass spectrum of NBD-Bu

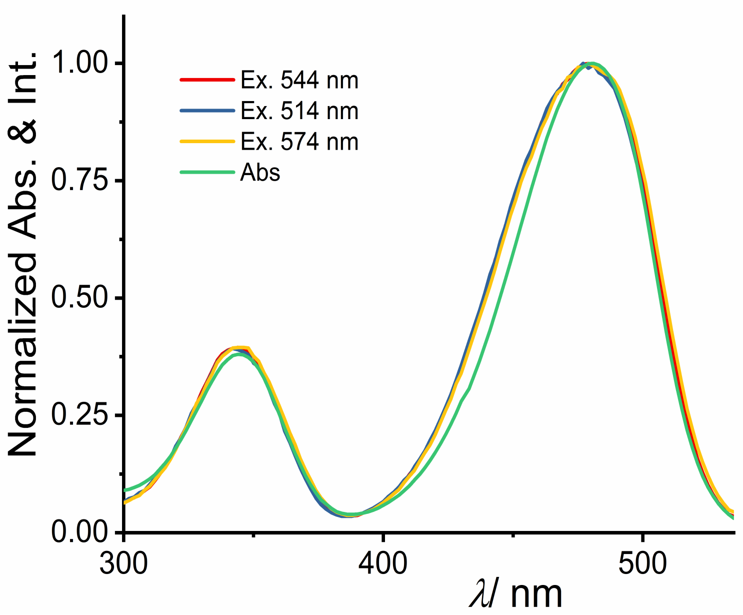

Fig. S4: Testing the optical purity of NBD-Bu using UV-Vis. absorption and excitation spectra in water (~pH 7.0).

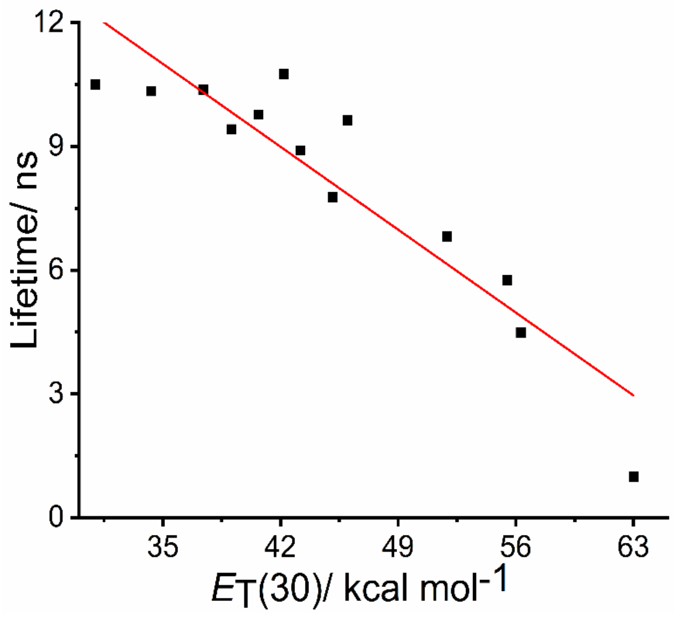

Fig. S5: The linear variation of the lifetime of NBD-Bu (10 μM) with solvent polarity parameter, $R^2 = 0.85$

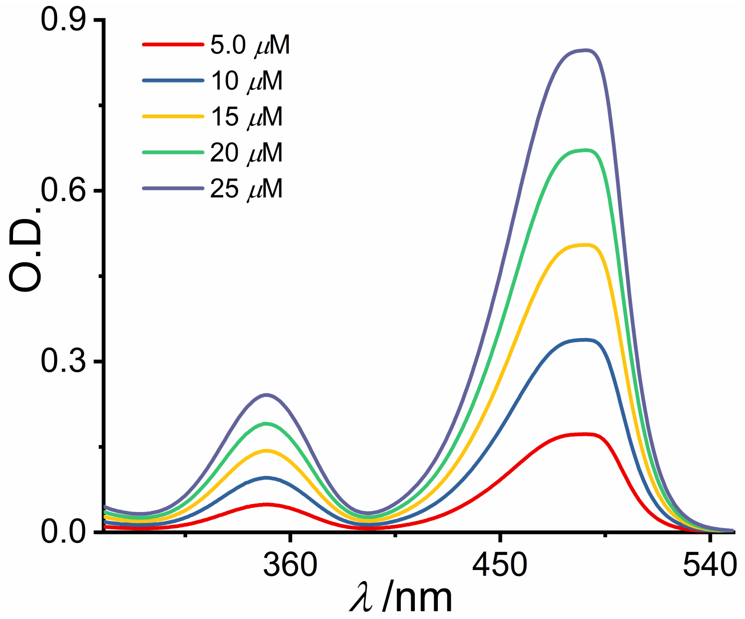

Fig. S6: Concentration-dependent absorption spectra of NBD-Bu in an aqueous medium (~pH 7.0)

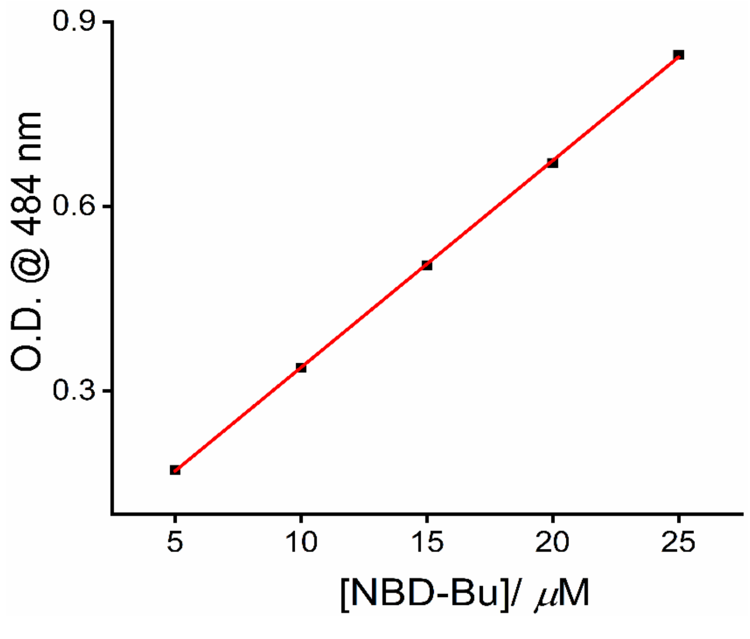

Fig. S7: The plot of optical density (O.D.) against increasing concentration of NBD-Bu at 484 nm (R^2= 0.99)

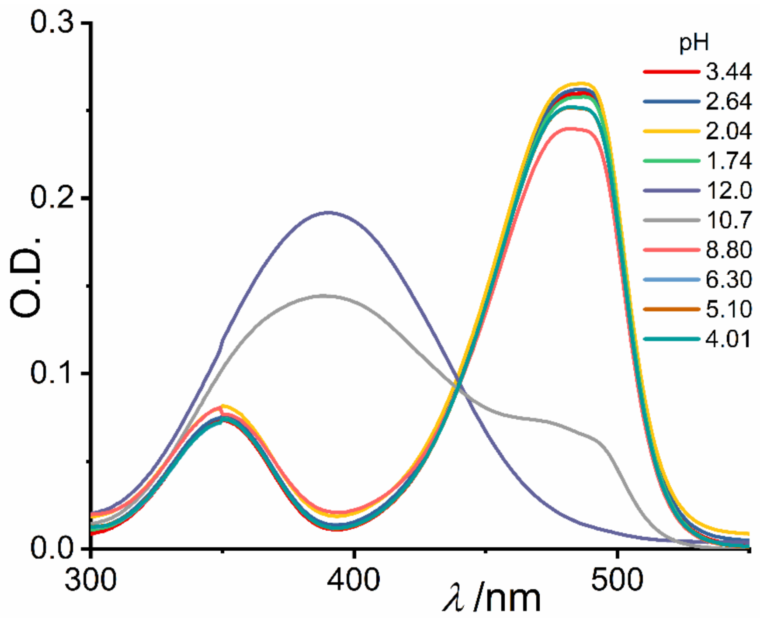

Fig. S8: pH titration of NBD-Bu (10 μM)

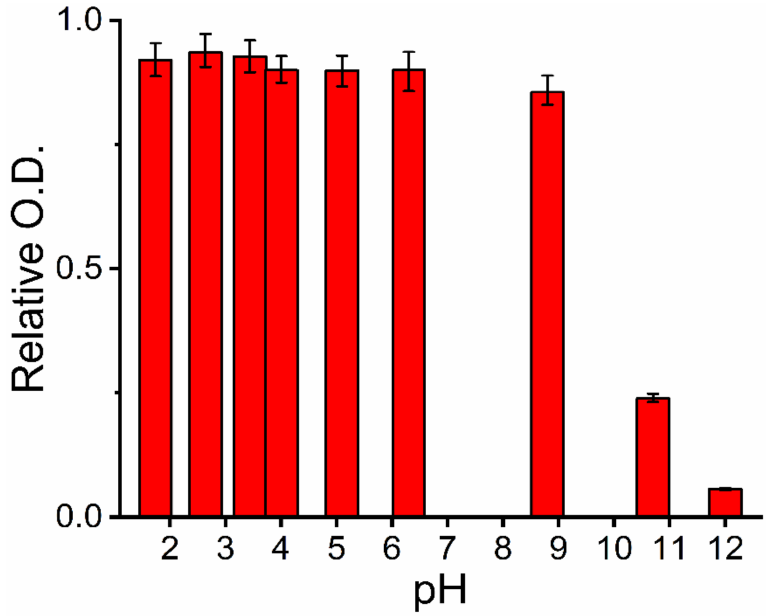

Fig. S9: The absorbance of NBD-Bu (10 μM) measured at different pH

Table S1: Solvent-dependent photophysical properties of NBD-Bu

Solvent	λ_{abs}/nm	λ_{em}/nm	Average lifetime/ ns	Rel. Quantum Yield[a]	$E_T(30)$[1]
Acetone	462	528	10.8	0.85	42.2
ACN	465	533	9.6	0.82	46.0
Benzene	453	520	10.3	1.135	34.3
Chloroform	452	527	9.4	1.05	39.1
DCM	456	523	9.8	0.65	40.7
DMSO	481	544	7.8	0.66	45.1
DMF	473	538	8.9	0.86	43.2
Ethanol	466	536	6.8	0.62	51.9
Ethylene glycol	476	552	4.5	0.30	56.3
Hexane	448	520	10.5	0.64	31.0
Methanol	467	539	5.7	0.35	55.5
THF	461	524	10.4	0.87	37.4
Water (PBS)	484	565	1.0	0.06	63
BSA (100 μM, PBS)	484	535	6.1	0.153	~42 (polarity is like acetone)

[a]Quantum yields were measured w.r.t. fluorescein dye in 0.1(N) NaOH.[2]

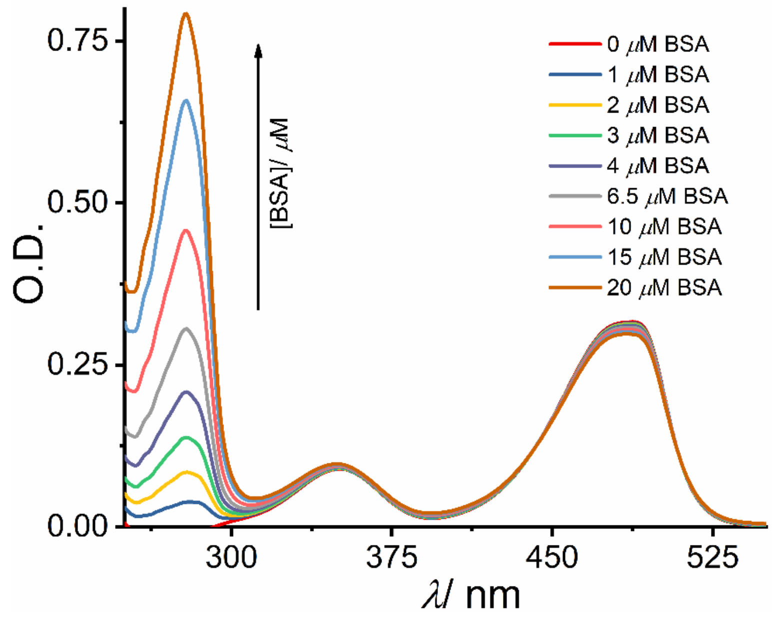

Fig. S10: UV-Vis. titration of NBD-Bu (10 μM) with increasing BSA concentration in PBS

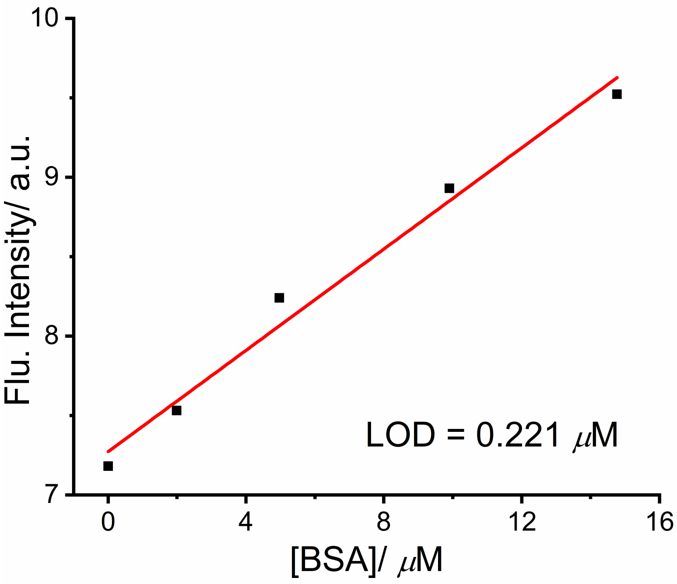

Fig. S11: Estimation of the limit of detection (LOD, 3σ/slope) from the calibration curve with BSA concentration ranging from 0-15 μM, R^2= 0.98

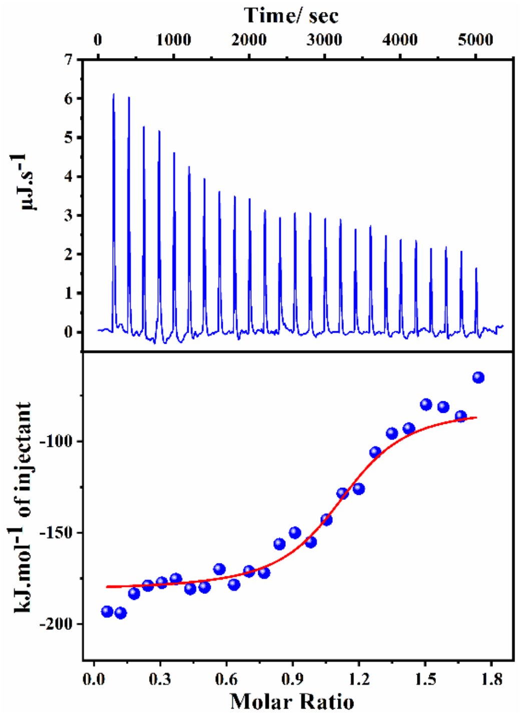

Fig. S12: Raw data of integrated heat change profile of BSA after binding to NBD-Bu; the fitted ITC plot shows the 1:1 binding stoichiometry between BSA and NBD-Bu.

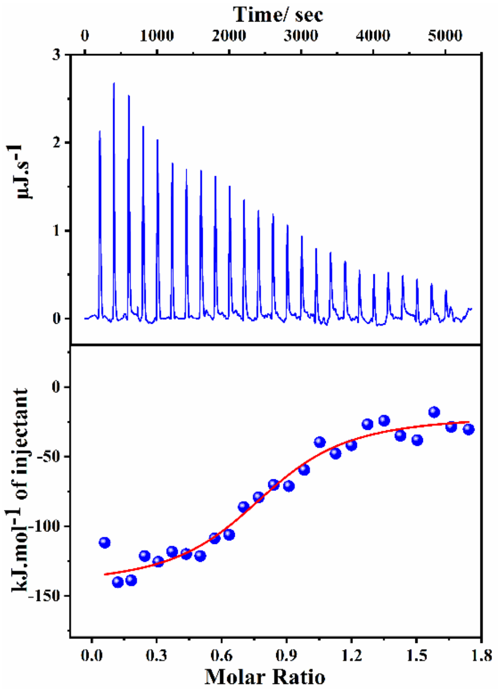

Fig. S13: Raw data of integrated heat change profile of BSA after binding to ibuprofen and the fitted ITC plot shows the 1:1 binding stoichiometry.

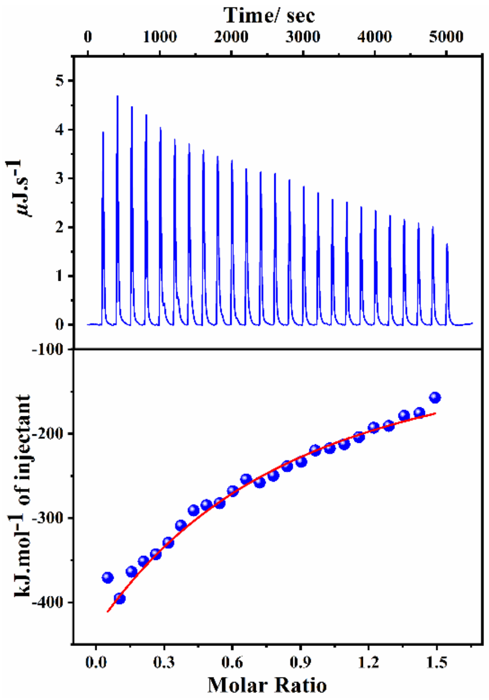

Fig. S14: Raw data of integrated heat change profile of BSA bound ibuprofen with NBD-Bu as an injectant

Table S2: Thermodynamic parameters obtained from ITC measurements

System	n	ΔH (kJ.mol^{-1})	ΔS (J.K.mol^{-1})	ΔG (kJ.mol^{-1})	This study: K_a (M^{-1})	Literature[3] K_a (M^{-1})
BSA-NBD-Bu	1.05	-100	-218	-35.03	$1.30x10^6$	--
BSA-Ibu	1.15	-200	-661	-3.02	$3.37x10^5$	$3.03x10^5$

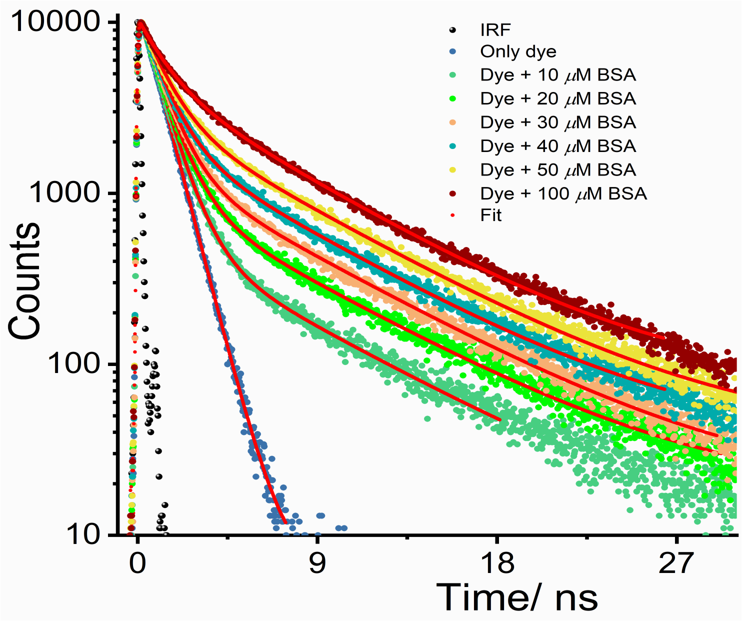

Fig. S15: Increase in the fluorescence lifetime of NBD-Bu with increasing BSA concentration in PBS buffer.

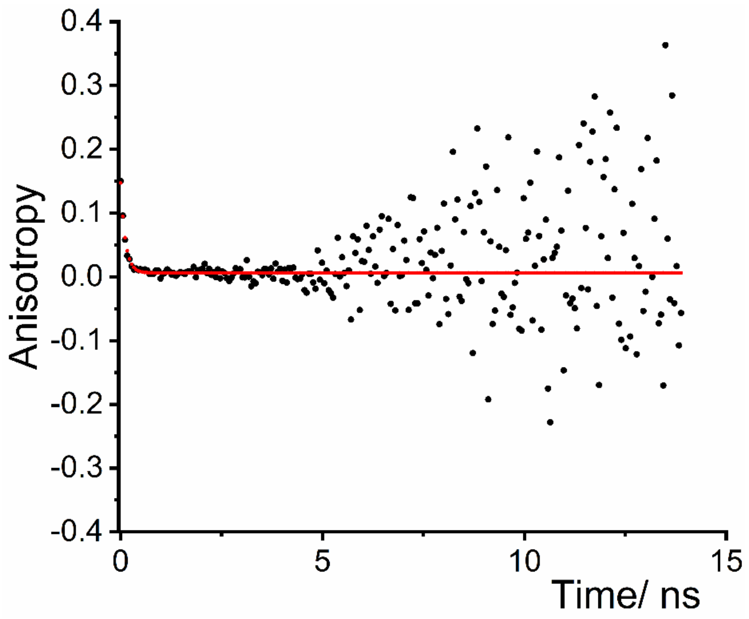

Fig. S16: Fluorescence anisotropy of 10 μM NBD-Bu in PBS (pH 7.4)

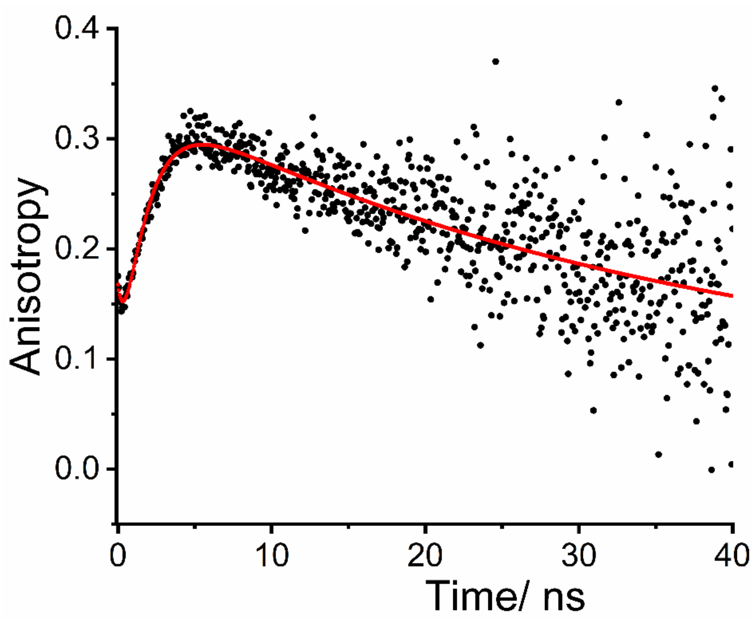

Fig. S17: Fluorescence anisotropy of 10 μM NBD-Bu in presence of 100 μM BSA in PBS (pH 7.4)

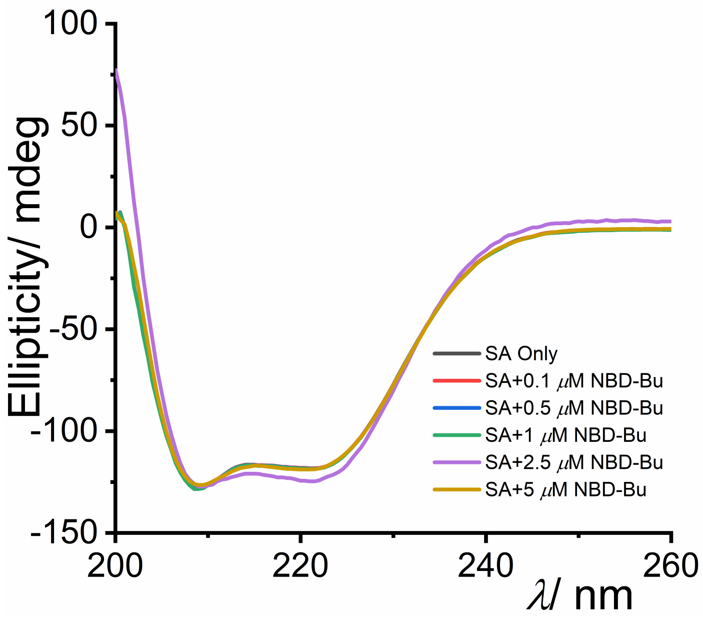

Fig. S18: Circular dichroism spectra of BSA (5 μM) with increasing concentration of NBD-Bu in PBS

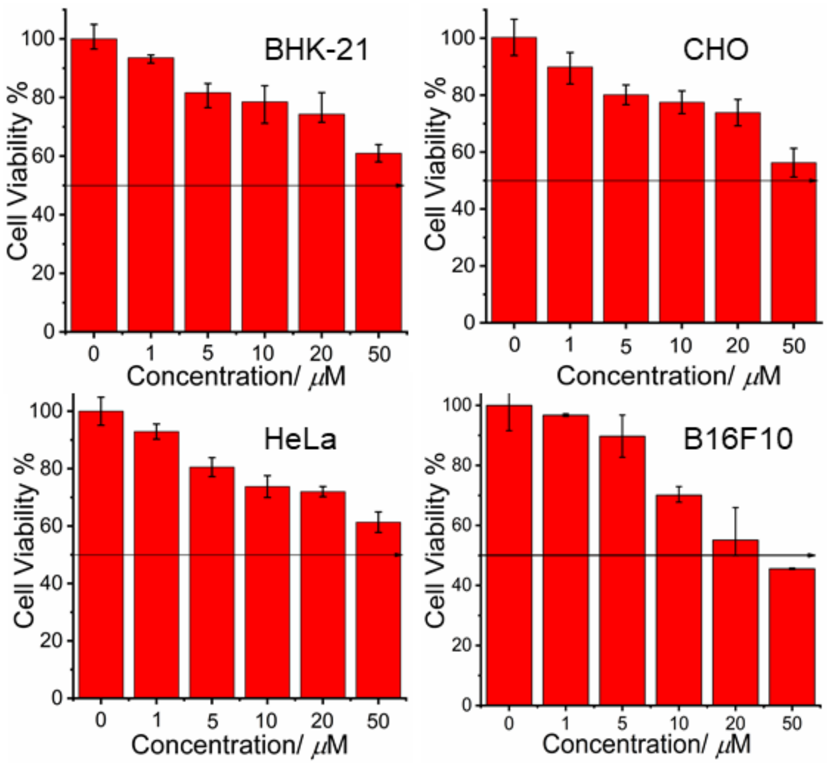

Fig. S19: MTT assay to assess the cell viability of BHK-21, CHO, HeLa and B16F10 cells against the different concentration of NBD-Bu.

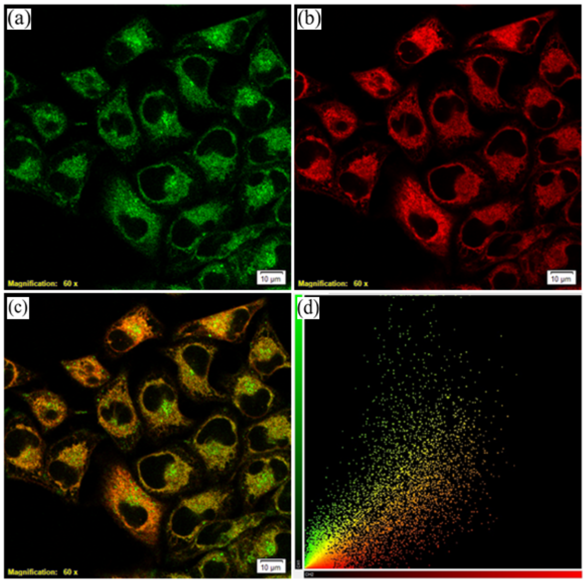

Fig. S20: HeLa cells stained with (a) 5 μM NBD-Bu, (b) 300 nM of ER tracker Red, (c) merge image of (a) and (b), (d) scatter plot showing Pearson's correlation coefficient of 0.81± 0.04.

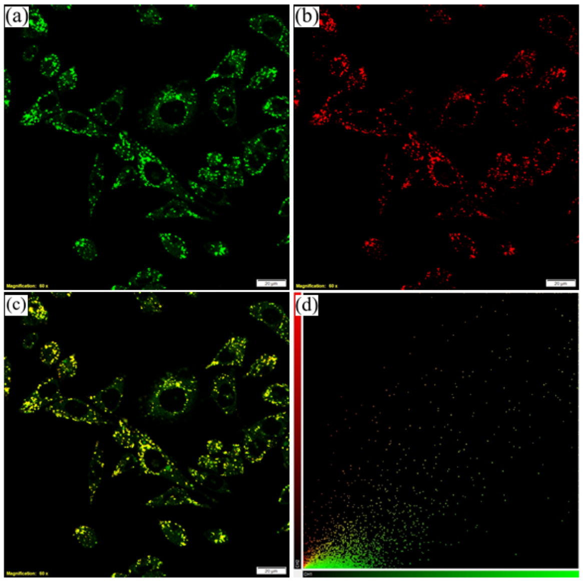

Fig. S21: HeLa cells stained with (a) 5 μM NBD-Bu, (b) 300 nM of Lysotracker Red, (c) merge image of (a) and (b), (d) scatter plot showing Pearson's correlation coefficient of 0.72± 0.03.

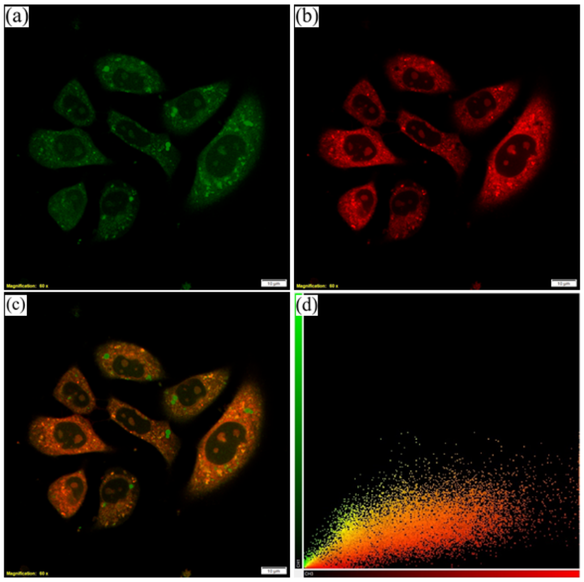

Fig. S22: HeLa cells stained with (a) 5 μM NBD-Bu, (b) 300 nM of Mitotracker Red, (c) merge image of (a) and (b), (d) scatter plot showing Pearson's correlation coefficient of 0.68± 0.02.

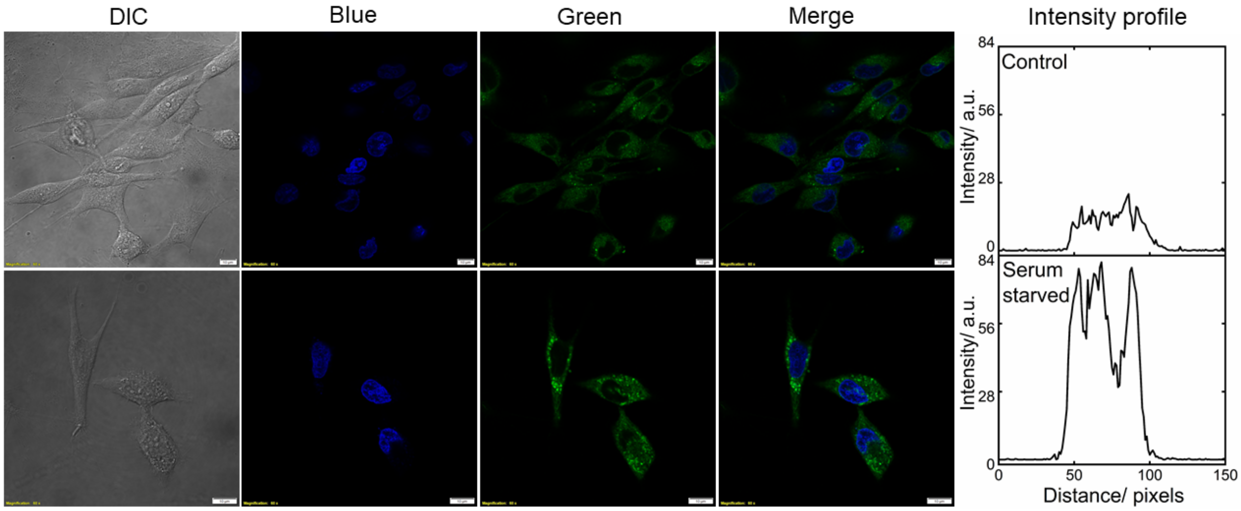

Fig. S23: Live-cell confocal microscopy imaging of well-fed and serum-starved BHK-21 cells

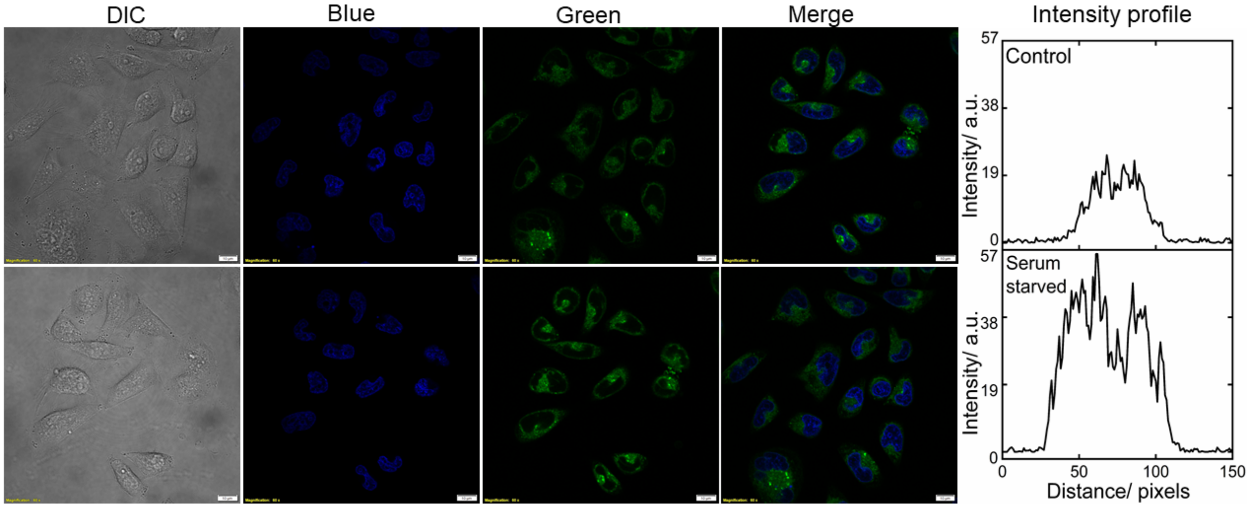

Fig. S24: Live-cell confocal microscopy imaging of well-fed and serum-starved HeLa cells

References: [1] C. Reichardt, T. Welton, Solvents and Solvent Effects in Organic Chemistry, John Wiley & Sons, Hoboken, 2011.

[2] R. Sjoback, J. Nygren, M. Kubista, Spectrochim. Acta A, 51 (1995) L7-L21.

[3] C. Ràfols, S. Zarza, E. Bosch, Talanta, *130* (2014), 241-250
